# Integrative Analysis of Histone Deacetylases Reveals the Potential Role and Prognostic Value of HDAC7 in Hepatocellular Carcinoma

**DOI:** 10.7150/jca.112983

**Published:** 2025-07-24

**Authors:** Changjiang Yang, Zhenze Yu, Ranran Wang, Meijing Zhang, Zihan Zhao

**Affiliations:** 1 Peking University Aerospace School of Clinical Medicine, Aerospace Center Hospital, China.; 2 Department of Gastroenterological surgery, Peking University People's Hospital, China.

**Keywords:** Histone deacetylases, Hepatocellular carcinoma. HDAC7, prognosis.

## Abstract

Hepatocellular carcinoma (HCC) represents a complex malignancy characterized by molecular mechanisms that remain incompletely elucidated, underscoring the necessity for identifying genetic markers. Members of the histone deacetylase (HDAC) family are integral to epigenetic regulation, influencing chromatin architecture and transcriptional activity, thereby modulating gene expression within eukaryotic cells. Dysregulation of HDACs has been implicated in the initiation of aberrant transcriptional programs, contributing to the oncogenesis and progression of various cancers, including HCC. Despite the extensive HDAC family, the specific roles of individual members in HCC are not well-defined. This study utilizes data derived from clinical specimens and comprehensive databases to demonstrate that histone deacetylases, particularly HDAC7, exhibit differential expression in HCC samples, which correlates with significant variations in prognosis and pathological staging. Furthermore, functional enrichment analyses of HDAC7 and its co-expressed genes indicate that HDAC7 may act as an oncogene by modulating the expression of genes associated with key tumorigenic pathways. The analysis of immune cell infiltration further elucidates the association between HDAC7 expression levels and various immune cell types. In summary, HDAC7 emerges as a potential biomarker for the prognosis and diagnosis of HCC, providing significant insights for future research and therapeutic strategies.

## Introduction

Hepatocellular carcinoma (HCC) represents the main form of primary liver cancer and constitutes a significant healthcare challenge 1. HCC stands as the fourth most lethal cancer-related disease worldwide, with projections indicating that its mortality rate will increase over the next ten years 2. Key risk factors linked to this type of liver cancer include infections with hepatitis B and C, non-alcoholic fatty liver disease, and liver issues related to alcohol consumption 3. Although there are many therapeutic approaches for HCC today, the heterogeneity of this condition presents substantial challenges in diagnosing and treating it at advanced stages 4. Identifying biomarkers is essential for overcoming these obstacles and offers hope for early detection, accurate diagnosis, and tailored treatment strategies.

Histone deacetylases (HDACs), histone methyltransferases (HMTs), and DNA methyltransferases (DNMTs) serve essential functions in regulating chromatin structure. HDACs, in particular, are responsible for removing acetyl groups from lysine residues on both histones and non-histones, influencing a wide range of cellular activities 5. Since modifications to histones are key to maintaining chromatin architecture and regulating gene expression, alterations in histone acetylation are markedly linked to cancer progression. Additionally, numerous investigations have identified abnormal HDAC expression in various types of human cancers. Current research has demonstrated that elevated HDAC levels potentially correlate with advanced stages of disease and unfavorable patient outcomes. For example, Sudo T et al. suggested that HDAC1 level is linked to gastric cancer 6, and Rettig I et al. established HDAC8's connection to neuroblastoma development 7. As an important member of the HDAC family, HDAC7 has been found in earlier investigations to be associated with various inflammatory diseases and cancers 8. Currently, there are also studies showing that HDAC7 can act as an epigenetic modifier and a non-deacetylase to participate in multiple pathways and serve a pivotal function in liver inflammation and liver fibrosis 9, 10. However, at present, the specific role of the HDAC family in HCC remains unclear.

Our study analyzed the data in the TCGA and GEO databases and found that the expressions of multiple genes in the HDAC family were all different between HCC and normal samples. In particular, HDAC7 had significant changes in aspects such as prognosis, pathological staging, and immune infiltration. We conducted additional investigations into the pathways and functions of genes that show elevated and distinct expression patterns linked to HDAC7 and elucidated its operational mechanisms within HCC. Furthermore, we examined the link between HDAC7 mRNA levels and the immune microenvironment of tumors to deepen our comprehension of HDAC7's influence on anti-tumor immunity and HCC development. Our findings ultimately revealed that HDAC7 shows promise as a novel immunological indicator for future HCC therapeutic approaches.

## Methods and materials

### Acquisition and processing of data

We acquired RNA-seq data and corresponding clinical information from hepatocellular carcinoma (HCC) tumors and adjacent normal tissues through The Cancer Genome Atlas (TCGA; https://cancergenome.nih.gov/). Samples were selected using the following criteria: Inclusion: Histopathologically confirmed HCC; Complete clinical annotations, including TNM stage, survival status and follow-up duration. Exclusion: Patients with a history of other primary malignancies (e.g., colorectal, lung cancer); Missing data on key parameters (e.g., tumor grade, metastasis status); Preoperative receipt of chemotherapy, radiotherapy, or targeted therapy.

### Patients and clinical samples

Shanghai Outdo Biotech Company (Shanghai, China) supplied an HCC tissue microarray (HLivH150CS06) with 75 paired tumors and normal tissues, approved by their Ethics Committee (ID: SHYJS-CP-230701). And additional quality control was applied: Inclusion: Well-characterized tumor and adjacent normal tissues with clear histopathological diagnosis; Exclusion: Samples with severe autolysis, necrosis, or suboptimal fixation; Cases complicated by decompensated cirrhosis, autoimmune liver disease, or active viral hepatitis; Patients with systemic diseases (e.g., HIV infection, severe renal failure) potentially influencing epigenetic profiles.

### Immunohistochemistry (IHC) staining

Following paraffin embedding of HCC and normal tissue sections, specimens underwent dimethylbenzene dewaxing and ethanol rehydration. Antigen recovery proceeded via sodium citrate solution with microwave treatment at 95 °C. A 3% H2O2 solution blocked endogenous peroxidase activity for 10 minutes. Subsequently, specimens were submerged in a blocking solution and 10% fetal bovine serum for one hour. The samples were treated with primary antibody HDAC7 (1:100, CST #33418) at 4 °C overnight, succeeded by exposure to an HRP-labeled anti-rabbit secondary antibody in darkness. DAB visualization revealed immunostaining, while hematoxylin provided counterstaining before dehydration and mounting. lHC result interpretation criteria: independently scored by two pathologists, with the following scoring rules: A is scored based on the number of chromogenic cells: 1 point for positive cells less than 1/3, 2 points for positive cells from 1/3 to 2/3, and3 points for positive cells greater than 2/3; B is scored based on the depth of cell color: no positive reaction cells and 0 points, light yellow is 1 point, brown yellow is 2 points, and brown is 3points. Rating=A + B. A score of no more than 3 indicates low expression. And a score of more than 3 indicates high expression.

### Survival analysis

Survival analyses were executed employing the Kaplan - Meier (KM) approach and log - rank examination. The mean value of HDAC7 expression was utilized as the threshold. Cox regression assessments were executed to evaluate the link between patient outcomes and clinical as well as pathological variables.

### Enrichment analysis

In this investigation, we investigated the biological function of HDAC in HCC. We carried out differential gene expression analyses (DEGs) on the low - and high - expression cohorts. The significance thresholds were set as |logFC| > 1 and FDR < 0.05. We used the ClusterProfiler tool in R (version 3.6.3) to execute functional enrichment evaluation of Gene Ontology (GO) and Kyoto Encyclopedia of Genes and Genomes (KEGG), as well as gene set enrichment analysis (GSEA). The GO analysis covered molecular functions (MFs), cellular components (CCs), and biological processes (BPs). We utilized GSEA to assesses the statistical significance and consistency of differences between two biological states based on a pre-defined gene set. For each phenotype, the enriched pathways were categorized using a normalized enrichment score (NES) and an adjusted p-value. The c2.cp.kegg.v2022.1.Hs.symbols.gmt [KEGG Pathway Database] served as the reference gene collection for the KEGG pathways. Moreover, c5.go.all.v2022.1.Hs.symbols.gmt [GO] (10561) was utilized as the reference gene collection for GO terms. When the adjusted p - value was < 0.05 and the false discovery rate (FDR) was < 0.25, the gene sets were markedly enriched.

### Immune infiltration analyses

We utilized the ESTIMATE algorithm to ascertain the immune and stromal scores of HCC. Spearman's correlation assessment was executed to examine the link between HDAC7 level and these immune cells. To assess the variations in immune cell infiltration levels between the low-expression cohort and the high-expression cohort, the Wilcoxon rank - sum test was implemented.

### Statistical analysis

Statistical data from TCGA were analyzed using R 4.2.1. The assessment of HDAC7 level variations between neoplastic and healthy specimens employed both Wilcoxon rank - sum and signed - rank evaluations. In addition, Welch's one-way ANOVA incorporating Bonferroni's post - hoc analysis (or the t - test) was applied to investigate possible associations between HDAC7 expression and clinicopathological characteristics. The research incorporated Pearson's chi - square evaluation to examine how diverse clinicopathological factors influenced HDAC7 expression. When chi - square test groups did not meet criteria of total sample size > 40 or theoretical frequency > 5, Fisher's exact probability method was applied. A K-M curve was constructed to ascertain HDAC7's predictive potential. Additionally, Cox regression examinations were executed to examine Overall Survival (OS) and Disease Specific Survival (DSS), seeking to establish the prognostic value of HDAC7 expression combined with additional clinicopathological parameters. The pROC package facilitated the Receiver Operating Characteristic (ROC) examination of HDAC7. The documented area under the curve (AUC) measurements, spanning 0.5 to 1.0, suggest discriminatory ability from 50% to 100%. Statistical relevance was developed through a two - tailed P - value of ≤ 0.05 across all analyses.

## Results

### The relationship between the HDAC gene family and HCC

**Figure 1:**
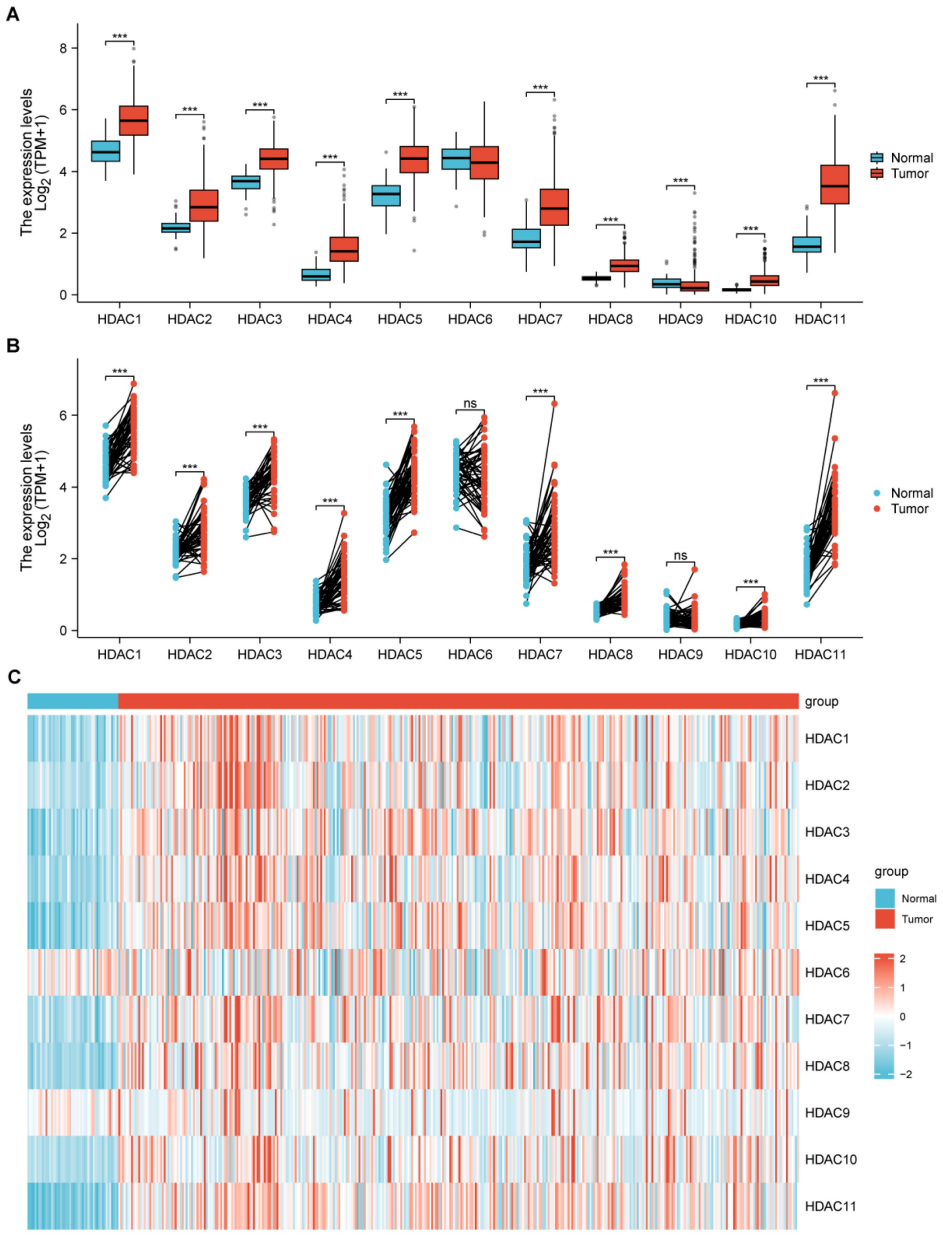
** The relationship between the HDAC gene family and HCC. (A, B)** TCGA analysis illustrated that partial HDAC mRNA expression was upregulated in HCC in comparison with normal samples. **(C)** Heatmap depicting the differential expression of HDAC genes between normal and tumor tissues. (ns represents no significance, * p < 0.05, ** p < 0.01, *** p < 0.001).

The research evaluated HDAC gene family level in HCC and normal tissues by using TCGA-based data. Multiple HDAC family members displayed distinct mRNA expression variations when comparing HCC to normal samples, with remarkable differences noticed in HDAC1, HDAC2, HDAC3, HDAC4, HDAC5, HDAC7, HDAC8, HDAC10, and HDAC11 (Figure 1A, B). Moreover, we demonstrated the distribution of the HDAC family in normal tissues and HCC tumor tissues through heatmaps, among which most HDACs had obvious differences in distribution (Figure 1C).

**Figure 2:**
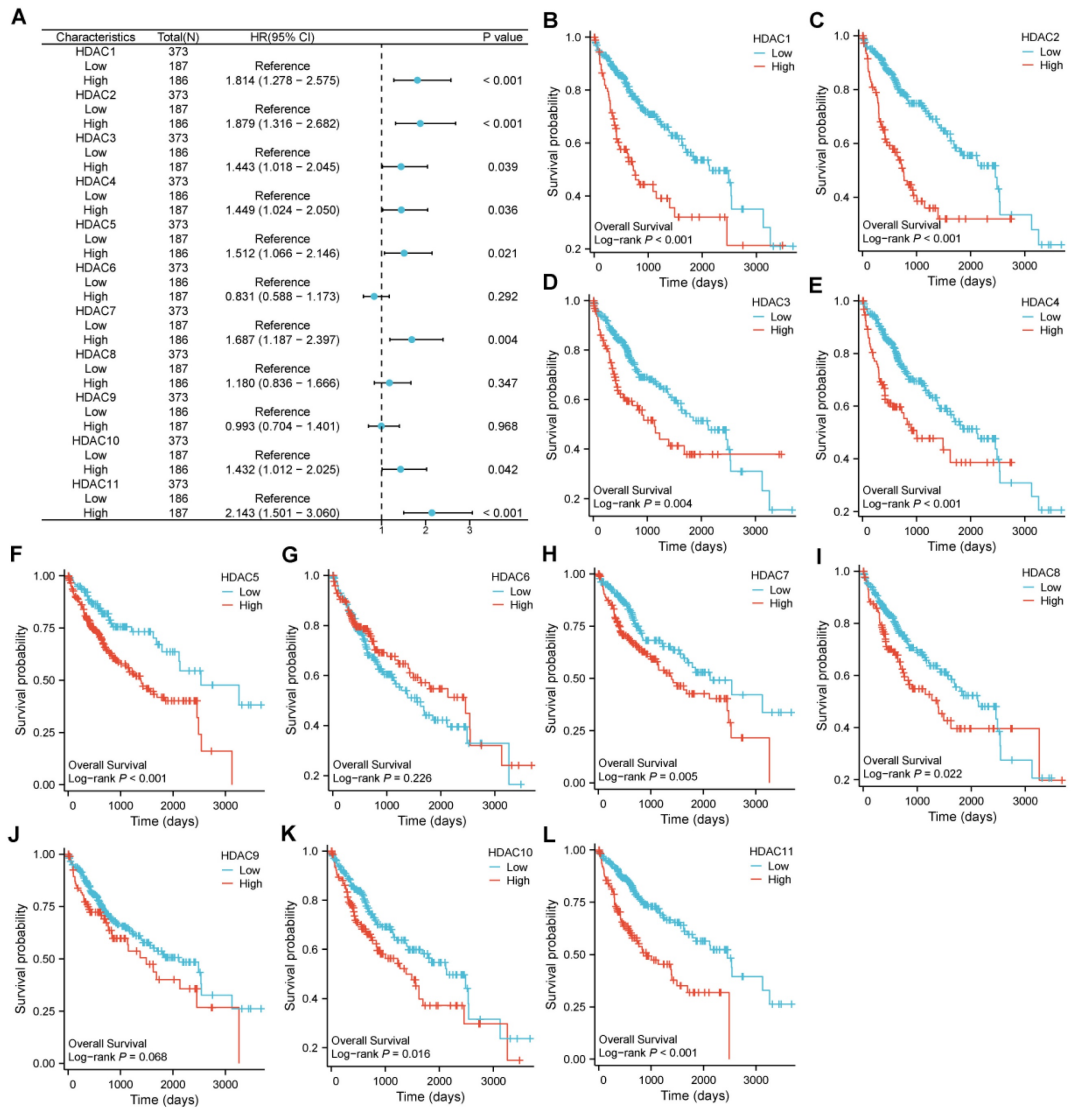
** The hazard ratio of OS for HDAC genes between low-expression and high-expression samples. (A)** Correlation of differential expression of HDAC genes in HCC. **(B-L)** The KM plots of OS for HCC patients according to the different HDAC expressions.

The cutoff point for categorizing HCC specimens into groups with low and high expression was developed using the median HDAC level. Significant variations in OS emerged between specimens with low and high expression for certain HDAC genes. Among them, the COX regression analysis indicated that HDAC1, HDAC2, HDAC3, HDAC4, HDAC5, HDAC7, HDAC10, and HDAC11 were involved (Figure 2A). Moreover, in the LOG-rank test, we found that in addition to the above-mentioned genes, HDAC8 also showed differences (Figure 2B-L).

**Figure 3:**
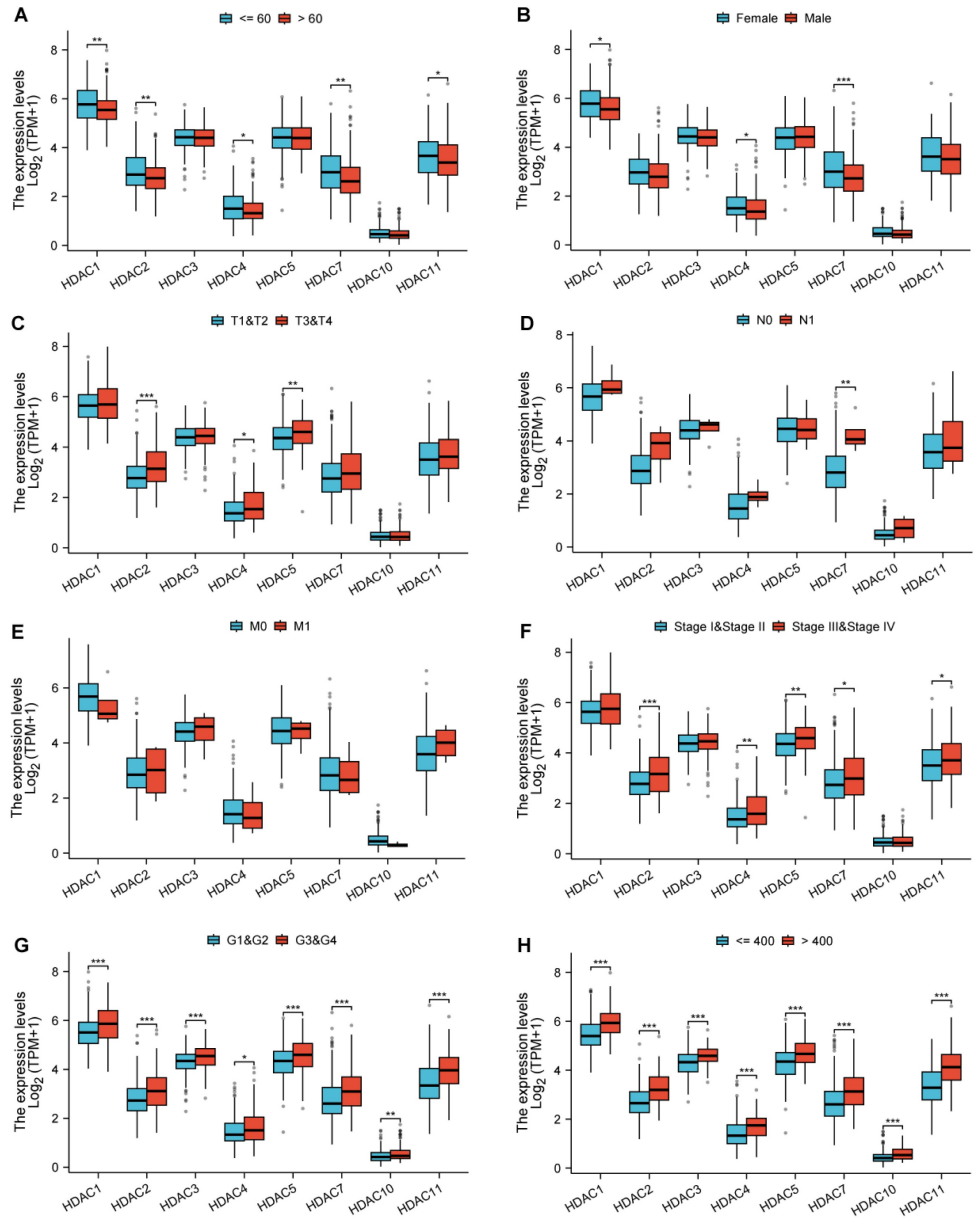
** Associations between HDAC expression and clinicopathologic characteristics as well as prognosis in HCC based on the TCGA database.** (Blank represents no significance, * p < 0.05, ** p < 0.01, *** p < 0.001).

We analyzed the association of the genes with statistically significant differences identified from the above-mentioned analysis with various clinical and pathological features. Among them, HDAC1, HDAC2, HDAC7, and HDAC11 showed differences in age distribution (Figure 3A); HDAC1 and HDAC7 showed differences in gender distribution (Figure 3B); HDAC2, HDAC4, and HDAC5 showed differences in pathological T-stage expression (Figure 3C); HDAC7 showed differences in pathological N-stage (Figure 3D); All HDAC genes have no statistical significance in pathological M-stage (Figure 3E); HDAC2, HDAC4, HDAC5, HDAC7, and HDAC11 showed differences in Pathologic stage (Figure 3F); and all genes showed statistically significant differences in other pathological analyses (Figure 3G,H).

**Figure 4:**
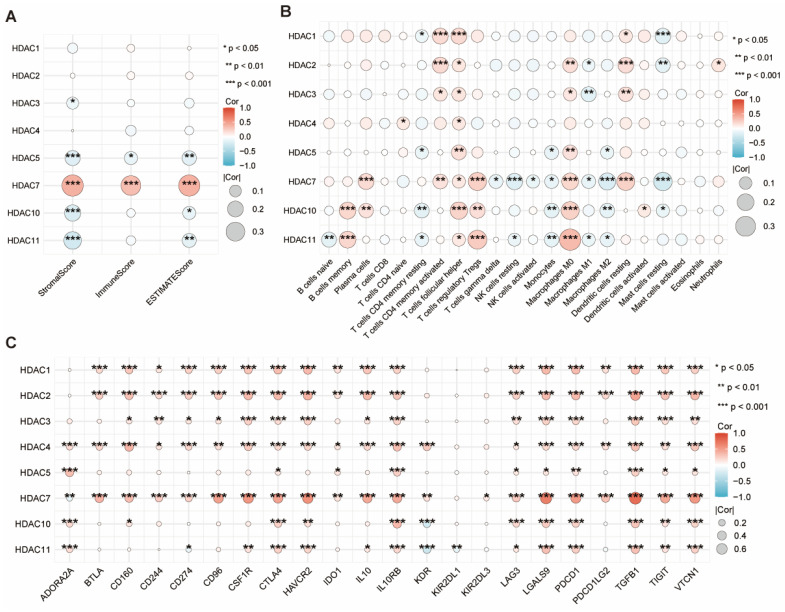
**(A)** The ESTIMATE algorithm was utilized to assess the link between diverse HDAC expressions with the ESTIMATE, stromal, and immune scores. **(B)** Correlation of different HDAC expression with immune infiltration in HCC. **(C)** Correlation between immunoinhibitory genes and different HDAC in HCC. (ns represents no significance, * p < 0.05, ** p < 0.01, *** p < 0.001).

We employed the ESTIMATE algorithm to obtain the immune, stromal, and ESTIMATE scores for HCC. We found that only HDAC5 and HDAC7 showed significant statistical significance in the three scores, with HDAC7 being the most prominent (Figure 4A). Subsequent examination explored the link between HDAC levels and immune cell infiltration levels in HCC, along with the tumor microenvironment (TME) characteristics. Among them, HDAC7 showed significant statistical significance in most immune cells (Figure 4B). Furthermore, we evaluated the link between HDAC level and various immune-regulatory genes. The outcomes suggested that HDAC7 expression demonstrated significant associations with numerous targets in the immune microenvironment (Figure 4C).

In summary, through the above analysis of the association between the HDAC family and HCC, our results suggest that an elevated level of HDAC7 might contribute to unfavorable outcomes in HCC patients, and this effect can be reflected in regulating immune infiltration.

### Potential involvement of HDAC7 in HCC malignant progression

**Figure 5:**
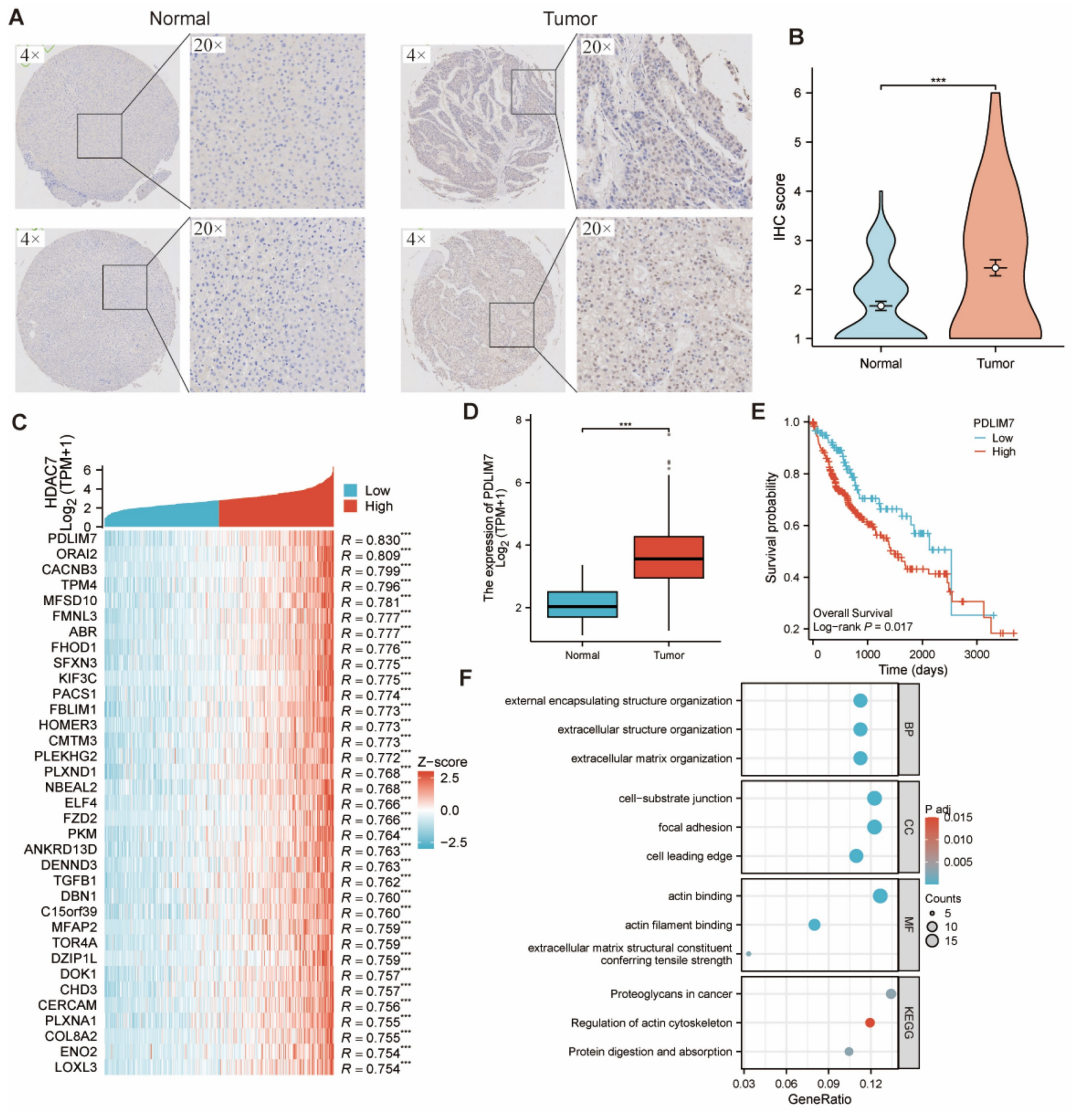
**(A, B)** HDAC7 protein expression was upregulated in HCC in comparison with normal samples, as shown by immunohistochemistry. **(C)** Heatmap illustrating the top 30 genes positively correlated with HDAC7 in HCC. Red represents high expression, and blue represents low expression. **(D)** PDLIM7 mRNA expression was upregulated in HCC compared with normal tissues based on TCGA. **(E)** The Kaplan-Meier curves show that HCC patients with a higher expression of PDLIM7 had a shorter OS time. (P=0.017) **(F)** GO and KEGG pathway analysis of HDAC7 co-expressed gene.

The IHC assay assessed HDAC7 levels in tumor and adjacent normal tissues from HCC specimens at Shanghai Outdo Biotech Company, revealing markedly higher HDAC7 IHC scores in tumors versus normal tissues. (Figure 5A, B). We compared the samples with high and low HDAC7 expression. Table 1 displayed an overview of HDAC7 protein associations with clinical and pathological parameters in HCC subjects. The logistics correlation analysis revealed substantial differences in HDAC7 quantities across pathologic stage, gender, age, histologic grade, and AFP levels (Table 2). Moreover, univariate and multivariate analyses indicated numerous clinical parameters exhibited strong associations with OS, encompassing pathologic T stage, pathologic M stage, and HDAC7 level (Table 3). In summary, HDAC7 level was closely linked to the clinical characteristics and prognosis of HCC patients.

We first identified the 30 most strongly associated genes showing significant correlation with HDAC7, as depicted in the heatmap, to investigate HDAC7's biological functions in HCC (Figure 5C). Among them, PDLIM7, as the related gene with the greatest difference, was found to have a significant statistical difference in its distribution between normal tissues and tumor cells (Figure 5D). Moreover, there was a difference in OS among patients with HCC (Figure 5E). Subsequently, we carried out GO and KEGG analyses on the genes that are co-expressed with HDAC7. The outcomes demonstrated that HDAC7 is involved in several BPs and MFs. It participates in the organization of external encapsulating structures, extracellular structures, and extracellular matrix. It also plays a part in cell-substrate junctions, focal adhesions, and forming the cell's leading edge. MFs are linked to actin binding and actin filament binding and serve as an extracellular matrix structural constituent that confers tensile strength.

Furthermore, the KEGG pathway investigation demonstrated that HDAC7 participates in pathways encompassing "Proteoglycans in cancer", "Regulation of actin cytoskeleton", and "Protein digestion and absorption". This indicates that HDAC7 may have diverse and important functions in cancer development and related BPs through its involvement in these pathways and molecular mechanisms (Figure 5F).

**Figure 6:**
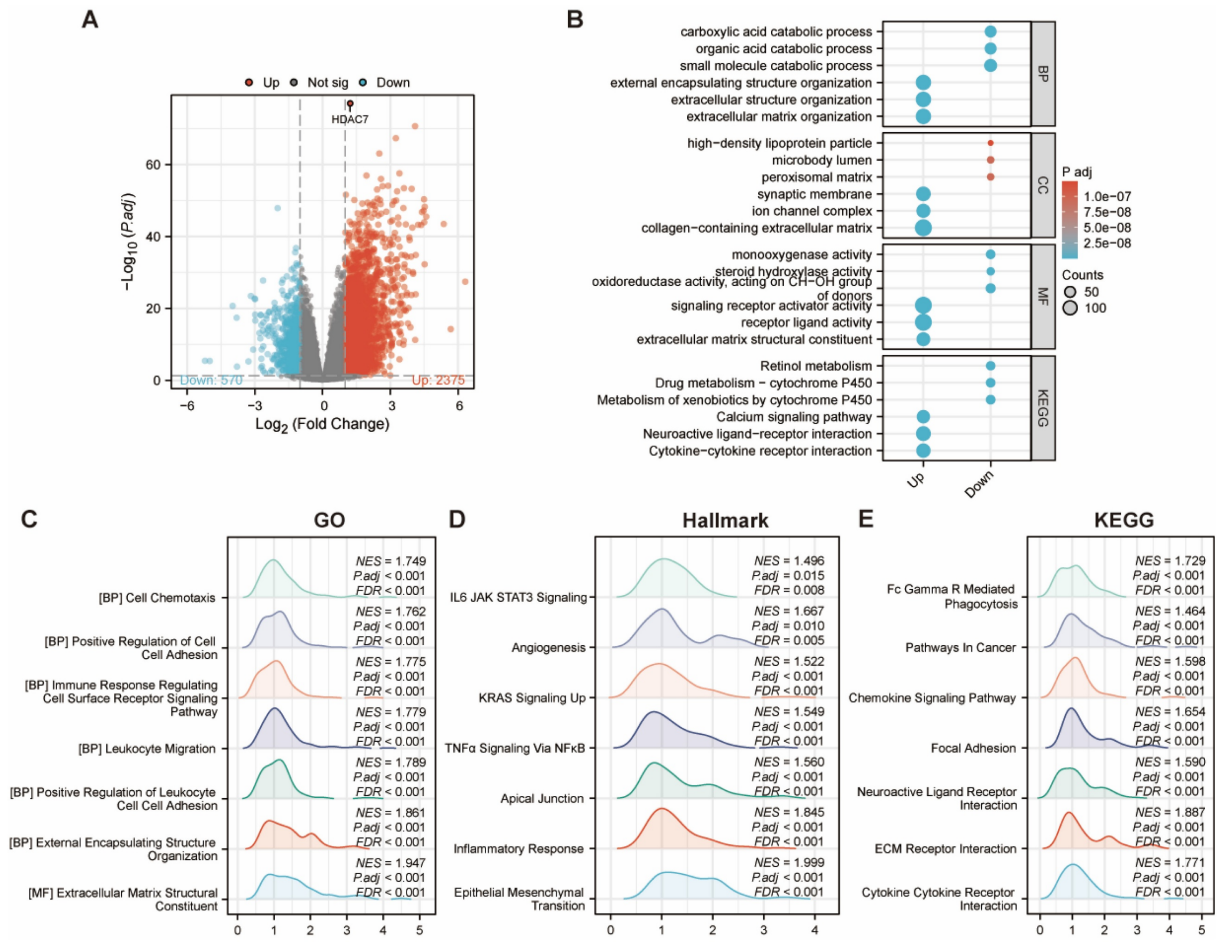
**(A)** Volcano plot depicting differentially expressed genes comparing HDAC7 high versus low expression cohorts in HCC. Elevated expression is indicated in red, while reduced expression appears in blue. **(B)** Functional enrichment analysis utilizing GO terms and KEGG pathways for the identified up- and down-regulated DEGs. **(C-E)** GSEA pathway analysis revealed associated signaling cascades linked to HDAC7.

We conducted a more in-depth analysis of the genes associated with HDAC7. Our findings indicated the presence of 2,945 differentially expressed genes (DEGs) when comparing the low-expression cohort with the high-expression cohort. Specifically, within the high-expression subgroup, 2,375 genes were elevated, while 570 genes were diminished (Figure 6A).

Following this, we proceeded with GO and KEGG pathway analyses. The bubble chart demonstrated that the upregulated DEGs were involved in several pathways, including external encapsulating structure organization, extracellular structure organization, extracellular matrix organization, synaptic membrane, ion channel complex, collagen-containing extracellular matrix, signaling receptor activator activity, receptor ligand activity, and extracellular matrix structural elements. Additionally, the KEGG pathway investigation indicated that HDAC7 exhibited crucial functions in pathways, including the Calcium signaling cascade, Neuroactive ligand-receptor communication, and Cytokine-cytokine receptor communication. This thorough examination yielded an essential understanding regarding the possible functions and mechanisms of HDAC7 and its associated genes, potentially advancing our knowledge of diverse BPs and disorders (Figure 6B). We subsequently utilized the GSEA enrichment examination to evaluate the HDAC7-associated genes. These enrichments revealed that HDAC7-associated differential genes in HCC participated in numerous BPs and pathways, comprising cell chemotaxis, immune response modulation, angiogenesis, and cancer-associated pathways (Figure 6C-E).

**Figure 7:**
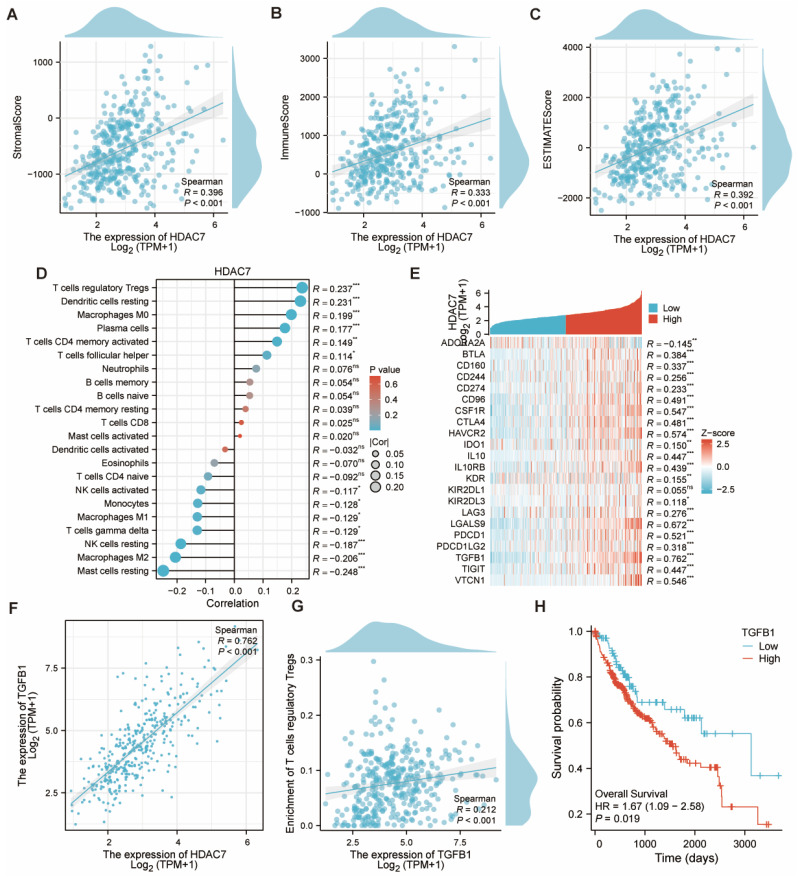
** The link between HDAC7 and immunological components in HCC. (A-C)** The ESTIMATE algorithm examined the link between HDAC7 level and the ESTIMATE, stromal, and immune scores. **(D)** Correlation of different HDAC7 expression with immune infiltration in HCC. **(E)** Heatmap illustrating the correlations between immunoinhibitory genes and HDAC7 in HCC. **(F)** Correlation between HDAC7 expression and TGFB1 in HCC. **(G)** Correlation between Tregs and TGFB1 in HCC. **(H)** The KM plots for OS for HCC patients according to the difference TGFB1 expressions. (ns represents no significance, * p < 0.05, ** p < 0.01, *** p < 0.001).

The immune, stromal, and ESTIMATE scores for HCC were calculated utilizing the ESTIMATE algorithm. Subjects exhibiting elevated HDAC7 expression displayed greater scores versus those with reduced HDAC7 levels, revealing a notable positive association (Figure 7A-C). Subsequently, we examined the links between HDAC7 and various immune cells. Several cell types, including T cells regulatory Tregs, Dendritic cells resting, and Macrophages M0, exhibited positive correlations, whereas NK cells resting, Macrophages M2, and Mast cells resting showed inverse relationships (Figure 7D). Moreover, we examined the association between HDAC7 and other immune-regulatory genes. Our research revealed that HDAC7 expression exhibited substantial connections with numerous targets in the immune microenvironment (Figure 7E). The analysis presented in Figure 5F revealed a robust association between transforming growth factor β1 (TGFB1) and HDAC7. A similar pattern emerged with T cells regulatory Tregs, demonstrating the strongest immune cell correlation (Figure 7G). Furthermore, KM analysis revealed that HCC patients with high TGFB1 expression demonstrated reduced OS duration (Figure 7H). This finding points to a possible mechanism explaining how enhanced HDAC7 expression might lead to unfavorable HCC outcomes. These observations also indicate HDAC7's potential involvement in regulating immune infiltration in HCC.

### Pan-cancer analysis of HDAC7

**Figure 8:**
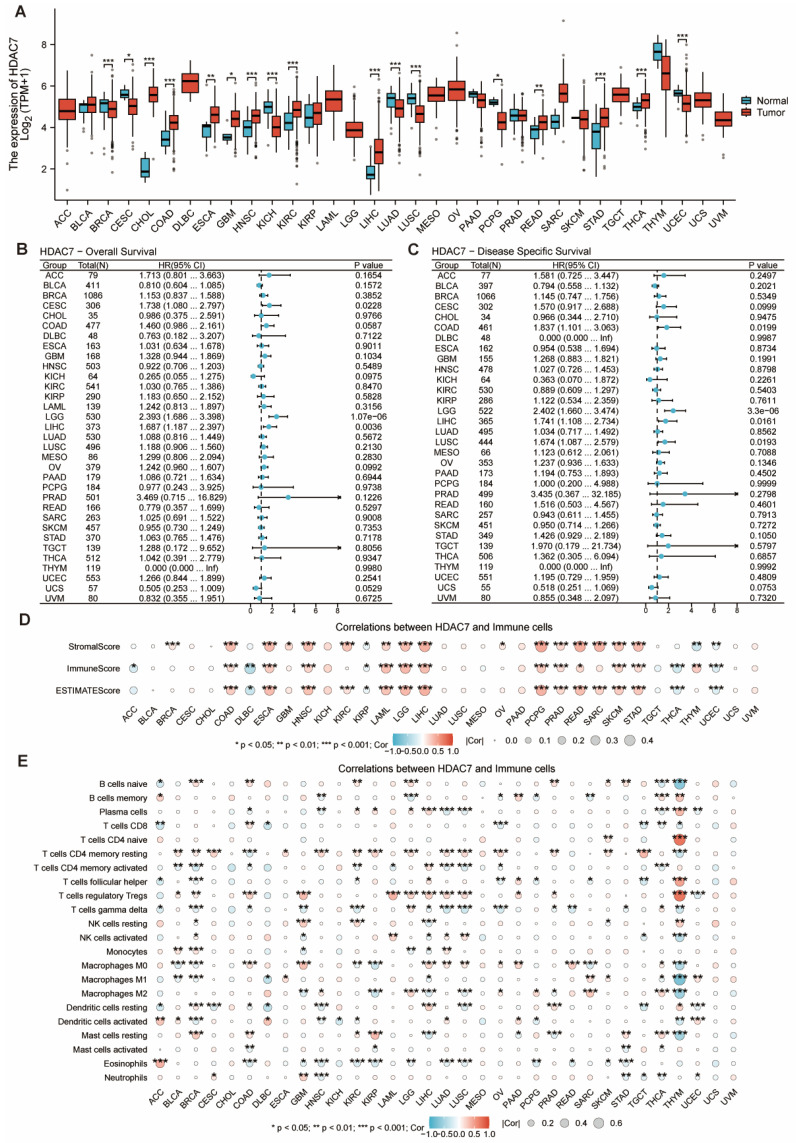
** The mRNA and HDAC7 level in pan-cancer. (A)** HDAC7 mRNA level in pan-cancer tissues versus normal tissues per the TCGA. **(B)** OS for pan-cancer according to the HDAC7 expressions. **(C)** DSS for pan-cancer according to the HDAC7 expressions. **(D)** The ESTIMATE algorithm was utilized to assess the link of HDAC7 level across pan-cancer with the ESTIMATE, stromal, and immune scores. **(E)** Correlation between immunoinhibitory genes and HDAC7 in pan-cancer. (ns represents no significance, * p < 0.05, ** p < 0.01, *** p < 0.001).

The investigation examined HDAC7 gene expression signatures across various human cancers and healthy tissues using TCGA-derived information. The HDAC7 mRNA expression quantities demonstrated marked differences between cancerous tissues and their normal counterparts, with substantial variations detected in breast invasive carcinoma (BRCA), cholangio carcinoma (CHOL), colon adenocarcinoma (COAD), head and neck squamous cell carcinoma (HNSC), kidney chromophobe (KICH), kidney renal clear cell carcinoma (KIRC), liver hepatocellular carcinoma (LIHC), lung adenocarcinoma (LUAD), lung squamous cell carcinoma (LUSC), stomach adenocarcinoma (STAD), thyroid carcinoma (THCA) and uterine corpus endometrial carcinoma (UCEC) (Figure 8A). Our research team investigated the connection between the HDAC7 gene and survival outcomes in various tumors. The findings revealed significant differences in OS and DSS of the HDAC7 gene between specimens showing low and high expression in certain malignancies, particularly brain lower grade glioma (LGG) and liver LIHC (Figure 8B, C). Additional evaluation was conducted on the association between HDAC7 expression in immune infiltration (Figure 8D) and immune cells (Figure 8E). The outcomes suggest that it has statistical significance in multiple tumors, which provides a direction for future research.

## Discussion

HCC represents a multifaceted disease characterized by cytokine changes that disrupt multiple signaling cascades, resulting in tumor formation, progression, and increased aggressiveness 1. A thorough understanding of the various developmental pathways associated with HCC is essential for effective detection, management, and potential alteration of the disease's clinical course. The next step involves pinpointing a novel biological marker linked to immune infiltrates, as well as discovering a new molecular pathway that could enhance the effectiveness of immunotherapy 11. HDAC is involved in multiple different stages of cancer. Its abnormal expression is associated with various malignant tumors, including HCC 12. Aberrant HDAC7 expression and genetic alterations are linked to the gravity and advancement of numerous ailments. This characteristic endows HDAC7 with the potential to serve as a biomarker and therapeutic target. Currently, five HDAC inhibitors, namely Vorinostat, Belinostat, Romidepsin, Tucidinostat, and Panobinostat, have obtained approval for treating lymphoma and multiple myeloma. In addition, a plethora of pan - inhibitors and selective inhibitors are being investigated in clinical trials 13. These trials aim to explore their efficacy in treating diverse cancers as well as non - cancerous conditions, such as inflammatory disorders, neurodegenerative afflictions, and muscular dystrophies. Given these circumstances, selectively targeting HDAC7 for treating cancer and other HDAC - associated diseases holds promise and appears to be a viable approach 14.

Initially, we analyzed the TCGA database to verify the differential distribution of the HDAC family in HCC. By integrating the correlations with clinical characteristics, prognosis, staging, and the immune environment, we ultimately identified HDAC7 as a protein with markedly differential expression. We validated the increased expression of HDAC7 in HCC by examining both database records and patient samples. The assessment of specimens with varying HDAC7 abundance demonstrated correlations with multiple clinical and pathological characteristics. Afterward, we delved deeper into the signaling pathways and mechanisms of HDAC7 in HCC. We examined the co-expressed genes of HDAC7. Among these, PDLIM7, the gene most markedly associated with HDAC7, also showed a worse prognosis in HCC patients. We carried out GO and KEGG pathway assessments for genes showing co-expression with HDAC7. The findings revealed that HDAC7 participates in various BPs, encompassing the organization of external and extracellular structures and cell - related junctions. MFs are associated with actin binding and serve as a structural component of the extracellular matrix. The KEGG pathway analysis further revealed its roles in pathways including "Proteoglycans in cancer", "Regulation of actin cytoskeleton", and "Protein digestion and absorption". This indicates that HDAC7 plays diverse and crucial roles in cancer development and related BPs through these pathways and molecular mechanisms. Using the same approach, we analyzed the differentially expressed genes of HDAC7 and combined them with GSEA. The findings indicated that the HDAC7-related differential genes in HCC participate in multiple BPs and cascades, encompassing cell chemotaxis, regulation of the immune response, angiogenesis, and cancer-related pathways.

Moreover, our investigation revealed links between HDAC7 level and various immune marker genes along with immune cell populations. Earlier investigations have highlighted the essential role of the immune environment in cancer progression 15. The complex TME serves a vital function in tumor growth and spread. Our investigation demonstrated that HDAC7 expression exhibited substantial connections with diverse immune cells, encompassing regulatory T cells (Tregs), macrophages, mast cells, dendritic cells, and natural killer (NK) cells. Importantly, we observed a substantial link between HDAC7 and the immune-suppression gene TGFB1. The TGFB1 gene produces the TGF-β protein, which serves as a key component in the TGF-β pathway connected to multiple cancers 16. Prior investigations have shown that TGFB1 promotes HCC advancement and progression. When TGFB1 is upregulated, it can suppress the body's natural anti - tumor defenses, thus facilitating tumorigenesis 16-18. Our findings additionally indicated that TGFB1 correlates with unfavorable outcomes in HCC patients.

Finally, through the method of pan-cancer analysis, we noted that HDAC7 level varies in multiple tumors other than HCC, and there are differences in the prognosis of some tumors. This association is also related to the immune environment. This provides ideas for future research on the role of HDAC7 expression in other tumors.

In summary, our research has initially established the connection between HDAC7 and HCC. By employing tissue samples and sequencing data from public databases, we probed into the expression of HDAC7 in HCC and its prognostic implications. Moreover, bioinformatics approaches were utilized to explore its underlying mechanisms. Nevertheless, our study primarily validated the role of HDAC7 in HCC through bioinformatics approaches. Further experimental validation is required to comprehensively elucidate the mechanisms by which HDAC7 contributes to HCC pathogenesis and progression. For instance, employing loss-of-function or gain-of-function models in HCC cell lines could help demonstrate that HDAC7 promotes the malignancy of HCC, like enhancing proliferation, migration, and invasion. Further basic and clinical investigations are essential to clarify the biological significance of HDAC7 and its importance in the onset and progression of HCC.

## Conclusion

The results of our study indicate that HDAC7 shows higher expression levels in HCC tissues. It is linked to an advanced stage of the disease and a poor prognosis in HCC patients. As a result, HDAC7 has the potential to be a useful prognostic marker for those with HCC. Both fundamental research and clinical investigations are essential to thoroughly understand the biological importance of HDAC7 in HCC. This in - depth exploration will provide new perspectives for the detection and management of this malignant tumor in upcoming years.

## Figures and Tables

**Table 1:** The association of HDAC7 expression with clinicopathological features in the TCGA cohort

Characteristics	Low expression of HDAC7	High expression of HDAC7	P value
n	115	122	
Pathologic T stage, n (%)			0.216
T1	64 (27%)	53 (22.4%)	
T2	24 (10.1%)	28 (11.8%)	
T3	24 (10.1%)	34 (14.3%)	
T4	3 (1.3%)	7 (3%)	
Pathologic N stage, n (%)			0.146
N0	115 (48.5%)	118 (49.8%)	
N1	0 (0%)	4 (1.7%)	
Pathologic M stage, n (%)			1.000
M0	113 (47.7%)	120 (50.6%)	
M1	2 (0.8%)	2 (0.8%)	
Pathologic stage, n (%)			0.119
Stage I	64 (27%)	51 (21.5%)	
Stage II	24 (10.1%)	26 (11%)	
Stage III	25 (10.5%)	42 (17.7%)	
Stage IV	2 (0.8%)	3 (1.3%)	
Gender, n (%)			0.053
Female	29 (12.2%)	45 (19%)	
Male	86 (36.3%)	77 (32.5%)	
Age, n (%)			0.006
<= 60	53 (22.4%)	78 (32.9%)	
> 60	62 (26.2%)	44 (18.6%)	
Histologic grade, n (%)			0.060
G1	17 (7.2%)	12 (5.1%)	
G2	57 (24.1%)	46 (19.4%)	
G3	36 (15.2%)	59 (24.9%)	
G4	5 (2.1%)	5 (2.1%)	

**Table 2:** Logistics association between HDAC7 expression and clinicopathologic features

Characteristics	Total (N)	OR (95% CI)	P value
Pathologic T stage (T3&T4 vs. T1&T2)	237	1.650 (0.931 - 2.923)	0.086
Pathologic N stage (N1 vs. N0)	237	78741292.6038 (0.000 - Inf)	0.995
Pathologic M stage (M1 vs. M0)	237	0.942 (0.130 - 6.798)	0.952
Pathologic stage (Stage III&Stage IV vs. Stage I&Stage II)	237	1.905 (1.081 - 3.357)	**0.026**
Gender (Male vs. Female)	237	0.577 (0.330 - 1.009)	0.054
Age (> 60 vs. <= 60)	237	0.482 (0.287 - 0.812)	**0.006**
Histologic grade (G3&G4 vs. G1&G2)	237	1.992 (1.182 - 3.355)	**0.010**

**Table 3:** The univariate and multivariate analyses of OS

Characteristics	Total(N)	Univariate analysis		Multivariate analysis	
		Hazard ratio (95% CI)	P value	Hazard ratio (95% CI)	P value
Pathologic T stage	237				
T1&T2	169	Reference		Reference	
T3&T4	68	3.164 (2.004 - 4.993)	**< 0.001**	2.146 (0.290 - 15.877)	0.455
Pathologic N stage	237				
N0	233	Reference			
N1	4	2.098 (0.513 - 8.584)	0.303		
Pathologic M stage	237				
M0	233	Reference		Reference	
M1	4	3.972 (1.245 - 12.673)	**0.020**	1.938 (0.592 - 6.340)	0.274
Pathologic stage	237				
Stage I&Stage II	165	Reference		Reference	
Stage III&Stage IV	72	3.144 (1.993 - 4.962)	**< 0.001**	1.429 (0.194 - 10.542)	0.726
Gender	237				
Female	74	Reference			
Male	163	0.761 (0.476 - 1.217)	0.255		
Age	237				
<= 60	131	Reference			
> 60	106	1.195 (0.758 - 1.883)	0.443		
Histologic grade	237				
G1&G2	132	Reference			
G3&G4	105	1.034 (0.655 - 1.632)	0.886		
HDAC7	237				
Low	115	Reference		Reference	
High	122	1.915 (1.195 - 3.070)	**0.007**	1.906 (1.181 - 3.075)	**0.008**
